# Ubiquitin-Mimicking Peptides Transfer Differentiates by E1 and E2 Enzymes

**DOI:** 10.1155/2018/6062520

**Published:** 2018-08-30

**Authors:** Bo Jin, Jiayue Wang, Xiangnan Liu, Shuai Fang, Bo Jiang, Kay Hofmann, Jun Yin, Bo Zhao

**Affiliations:** ^1^Engineering Research Center of Cell and Therapeutic Antibody, Ministry of Education, and School of Pharmacy, Shanghai Jiao Tong University, Shanghai 200240, China; ^2^Department of Hand and Foot Surgery, The Second Affiliated Hospital of Soochow University, Suzhou 215004, China; ^3^Institute for Genetics, University of Cologne, Cologne 50674, Germany; ^4^Department of Chemistry, Center for Diagnostics and Therapeutics, Georgia State University, Atlanta 30303, USA

## Abstract

Ubiquitin and ubiquitin like proteins (UBLs) play key roles in eukaryotes. These proteins are attached to their target proteins through an E1-E2-E3 cascade and modify the functions of these proteins. Since the discovery of ubiquitin, several UBLs have been identified, including Nedd8, SUMO, ISG15, and Atg8. Ubiquitin and UBLs share a similar three-dimensional structure: *β*-grasp fold and an X-X-[R/A/E/K]-X-X-[G/X]-G motif at the C-terminus. We have previously reported that ubiquitin, Nedd8, and SUMO mimicking peptides which all contain the conserved motif X-X-[R/A/E/K]-X-X-[G/X]-G still retained their reactivity toward their corresponding E1, E2, and E3 enzymes. In our current study, we investigated whether such C-terminal peptides could still be transferred onto related pathway enzymes to probe the function of these enzymes when they are fused with a protein. By bioinformatic search of protein databases, we selected eight proteins carrying the X-X-[R/A/E/K]-X-X-[G/X]-G motif at the C-terminus of the *β*-grasp fold. We synthesized the C-terminal sequences of these candidates as short peptides and found that three of them showed significant reactivity with the ubiquitin E1 enzyme Ube1. We next fused the three reactive short peptides to three different protein frames, including their respective native protein frames, a ubiquitin frame and a peptidyl carrier protein (PCP) frame, and measured the reactivities of these peptide-fused proteins with Ube1. Peptide-fused proteins on ubiquitin and PCP frames showed obvious reactivity with Ube1. However, when we measured E2 UbcH7 transfer, we found that the PCP-peptide fusions lost their reactivity with UbcH7. Taken together, these results suggested that the recognition of E2 enzymes with peptide-fused proteins depended not only on the C-terminal sequences of the ubiquitin-mimicking peptides, but also on the overall structures of the protein frames.

## 1. Introduction

Ubiquitination is one of the crucial protein posttranslational modifications involved in most important physiological processes including protein degradation, cell signaling, gene expression, cell survival and differentiation, innate and adaptive immunity, cell cycle, and tumorigenesis [1, 2]. Ubiquitin, a polypeptide with 76 amino acids, is attached on the cellular substrates through a sequential enzymatic transfer cascade including ubiquitin activating enzyme (E1), ubiquitin conjugating enzyme (E2), and ubiquitin ligase (E3) [2]. The human genome encodes 2 E1s, 40 E2s, and over 600 of E3s [3]. Meanwhile, a series of ubiquitin like proteins (UBLs) have been discovered in recent years, which modify their downstream targets in a similar manner as ubiquitin [4, 5]. Among them two UBLs, Nedd8 (or Rub1) and the small ubiquitin-related modifier (SUMO), are relatively well studied [6, 7]. Nedd8 is the closest relative of ubiquitin and Neddylation plays a key role in a range of human diseases [8, 9], whereas SUMOylation tightly is linked to ubiquitination and thereby protein degradation [10].

Ubiquitin and UBLs share the same three-dimensional core structure: the *β*-grasp fold and a similar C-terminal motif X-X-[R/A/E/K]-X-X-[G/X]-G, where X represents any amino acid. We have previously reported short free peptides derived from the C-terminal sequences of ubiquitin and ubiquitin mutants could form the thioester intermediates with E1, E2, and E3 enzymes as full length ubiquitin [11, 12]. Encouraged by these results, we continued to investigate whether proteins carrying such peptides as C-terminal fusion tags could still be recognized by ubiquitin pathway enzymes. We searched for proteins which contain both *β*-grasp fold structure and the X-X-[R/A/E/K-X-X-G/X-G] motif in the UNIPROT databases. Eight protein candidates were selected as the targets and the corresponding short peptides based on the motif were synthesized. ATP-PPi assay showed that three of the eight peptides could be activated by the E1 enzyme Ube1. We subsequently fused these reactive peptides to three different protein frames including native frames, a wild-type ubiquitin frame and a PCP frame. Peptides fused with wild-type ubiquitin frame and PCP frame could be transferred to E1 well. An interesting thing is that PCP-peptide fusions lost the reactivity with E2 enzymes although they could be transferred to E1. These results suggested that the recognition of E2 with protein peptide fusions depended not only on the sequences of these C-terminal ubiquitin-mimicking peptides, but also on the overall structures of the proteins fused with the peptides.

## 2. Materials and Methods

### 2.1. Peptides and Genes Synthesis

By using a generalized profile constructed from known ubiquitin like proteins [13], we searched the UNIPROT sequence database for proteins expected to contain a ubiquitin like *β*-grasp fold. The list was narrowed down by manually selecting those ubiquitin like domains that end on the motif X-X-[R/A/E/K]-X-X-[G/X]-G. Eight proteins (seven from human, one from Drosophila) were selected and the heptamer peptides derived from these proteins were ordered from EZBiolab (Indiana, USA). The encoding genes of candidates 1, 2, and 7 were ordered from Genscript (New Jersey, USA) with the NheI and NotI restriction sites at the N- and C- termini, respectively.

### 2.2. ATP-PPi Exchange Assays

Initial velocity of Ube1-activated peptides either in the free state or on different frames was followed by the ATP-PPi exchange assay. 50 uL reactions were set up containing varying concentrations of peptides, 0.05 uM Ube1, 50 mM Tris-Cl, pH7.5, 10 mM MgCl_2_, and 1 mM ATP. The reactions were initiated by adding of 1 mM [^32^P]pyrophosphate (4.6 Ci/mol). The reactions were incubated at room temperature and quenched at various time points by adding of 0.5 mL of a suspension of activated charcoal (1.6 % (w/v) charcoal, 0.1 M tetrasodium pyrophosphate, and 0.35 M perchloric acid) to each of the reactions. The charcoal was pelleted by centrifugation and each charcoal pellet was washed three times with 1 ml 2 % trichloroacetic acid. Finally the charcoal pellet was resuspended in 0.5 ml water and the suspension was added to 3.5 mL Ultima Gold LSC-cocktail (PerkinElmer). The radioactivity bound to charcoal was determined by liquid scintillation counting. To measure the kinetics of the peptides activation by Ube1, initial velocities of ATP-PPi exchange were determined with concentrations of peptides varying from 10 uM to 1000 uM. The kinetic data were fitted to the Michaelis-Menten equation with the data analysis software Origin.

### 2.3. Cloning of the Peptides on Different Frames

To clone the peptides on ubiquitin and PCP frames, the genes of peptide C1, C2, and C7 were amplified by PCR with the following primers (Bo 134: 5'-GGA GAT ATA GCT AGC GCG GAA CCT GAT TTA AC-3', Bo164: 5'-GTA CGT ATA GCGGCCGC TCA GCC ACC ACA CAG ACG CGG TTC CGA TCC GCC ACC GCC AG -3', Bo165: 5'-GTA CGT ATA GCGGCCGC TCA GCC ACC CAG TTG ACG CAG CAC CGA TCC GCC ACC GCC AG -3', Bo166: 5'-GTA CGT ATA GCGGCCGC TTA GCC ACC CAG CAT ACG ACC TGC CGA TCC GCC ACC GCC AG -3'). The PCR products were double digested by NheI and NotI and inserted to the vectors. pET 28a (+) plasmid with wild-type ubiquitin gene (for ubiquitin frame) and pET 28a (+) plasmid with PCP and linker gene (for PCP frame ) were double digested by the same NheI and NotI restriction sites as the cloning vectors.

### 2.4. Protein Expression and Purification

The pET expression plasmids were transformed into BL21(DE3)pLysS chemical competent cells (Invitrogen) and plated on the LB-agar plates with appropriate antibiotics. Protein expression and purification followed the protocol provided by the vendor of the pET expression system (Novagen) and the Ni-NTA agarose resin (Qiagen). Typically, BL21(DE3)pLysS cells were transformed with the pET vector containing the recombinant gene and plated on a LB-agar plate with corresponding antibiotics. A single colony of the transformed cells was inoculated into 5ml of LB medium and the culture was grown overnight at 37°C. The next day the culture was diluted into 1L LB medium and grown at 37°C until OD reaches 0.6~0.8. The culture was then induced for protein expression with the addition of 1M IPTG. The culture was grown at 16°C for 12-14 h before the cells were harvested. To purify the protein from the cell, cells were pelleted and resuspended in 10 ml lysis buffer (50mM Tris, 500mM NaCl, 5mM imidazole, pH 7.5). 10 *μ*l MgCl_2_ (2M) and 10 *μ*l CaCl_2_ (2M) were added to cell suspension. Lysosome (5mg/ml) was also added to the cell suspension to lyse the cell wall polysaccharides. After being left on ice for 1 h, the cell suspension was sonicated to lyse the cells. The lysate was centrifuged and the supernatant was bound to Ni-NTA resin for 2 h. The resin was washed with 20 ml lysis buffer twice and eluted with 5ml elution buffer (same as lysis buffer but with 250mM imidazole). About 100 mg protein could be obtained after purification.

### 2.5. Western-Blot of Peptide Loading onto E1 and E2 Enzymes

Figure 2(b): 5 uM ubiquitin or CP1, CP2, or CP7 with an HA-tag at their N-termini was incubated with 1 uM Ube1 in the presence of 1 mM ATP, 10 mM MgCl_2_, and 50 uM DTT in TBS buffer (20mM Tris HCl, 150mM NaCl, pH 7.5) for 1 hour at room temperature.  Figure 3(a): 5 uM wild-type ubiquitin or UBP1, UBP2, or UBP7 with an HA-tag at their N-termini was incubated with 1 uM Ube1, 1 uM UbcH7 in the presence of 1 mM ATP, 10 mM MgCl_2_, and 50 uM DTT in TBS buffer (pH 7.5) for 1 hour at room temperature.  Figure 3(b): 5 uM wild-type ubiquitin or UBP1, UBP2, or UBP7 with an HA-tag at their N-termini was incubated with 1 uM Ube1, 1 uM UbcH7 in the presence of 1 mM ATP, and 10 mM MgCl2 in TBS buffer (pH7.5) for 1 hour at room temperature. Figures 4(a) and 4(b): 5 uM PCP-peptide (P1, P2, P3, P4 or C1, C2, C7, PCP-peptide means PCP protein fused with a specific peptide sequence at the C-terminal tail) with an HA-tag at their N-termini was incubated with 1 uM Ube1, 1 uM UbcH7 in the presence of 1 mM ATP, 10 mM MgCl_2_, and 50 uM DTT in TBS buffer (pH 7.5) for 1 hour at room temperature.  Figure 5: 5 uM PCP-peptide (C1, C2, C7) with an HA-tag at their N-termini was incubated with 1 uM Ube1, 1 uM UbcH5a, or UbcH5b in the presence of 1 mM ATP, 10 mM MgCl_2_, and 50 uM DTT in TBS buffer (pH 7.5) for 1 hour at room temperature. 20 uL of the reaction mixture in all western-blot assays above were loaded on a gradient of acrylamide (from 4-15%) SDS-PAGE reducing gel (Bio-Rad). For nonreducing SDS-PAGE gel, there are no *β*-mercaptoethanol and DTT in the reactions. After electrophoresis, the protein bands were electroblotted onto a piece of 0.45 *μ*m polyvinylidene fluoride membrane (Bio-Rad). The membrane was blocked with 3% BSA (Sigma, pH 7, ≥98%) in TBS buffer (pH 7.5) for 1 hour followed by incubation with 3% BSA in TBS buffer (pH 7.5) containing 1:500 diluted anti-HA antibody (Santa Cruz Biotechnology, sc-57592) and 1:10,000 diluted anti-mouse horseradish peroxidase conjugate (Thermo Fisher Scientific, Cat MA5-15739-HRP) for 1 hour, respectively. The membrane was then washed 5 times by the TBS-T buffer (20mM Tris HCl, 150mM NaCl, 0.05% Tween 20, 0.05% Triton X-100, pH 7.5) and 5 times by the TBS buffer (pH7.5) followed by detection with the ECL luminescent detection kit (GE Healthcare).

## 3. Results

### 3.1. Search New Motifs

We previously reported that the C-terminal sequence of ubiquitin is crucial for the E1-E2-E3 transfer [11]. Ubiquitin mutants with a C-terminal X-X-[R]-X-X-[G/X]-G motif in substitution for the wild-type C-terminal sequence V-L-R-L-R-G-G were reactive with E1 and E2s. Similarly, studies have demonstrated that replacing the wild-type C-terminal motif of the UBLs Nedd8 (which is V-L-A-L-R-G-G) and SUMO (which is Y-Q-E-Q-T-G-G) by X-X-[A/E/]-X-X-G-G motif would not affect their ability to form the covalent thioester intermediates with their cognate E1 and E2 enzymes [14, 15]. These findings enabled us to confirm that the C-terminal Gly and R/A/E/K residues ahead of the last Gly residue are indispensable for the transfer of ubiquitin and UBLs. We first searched the UNIPROT sequence database for proteins expected to contain a *β*-grasp fold, since the *β*-grasp fold structure is found in all UBLs [16]. The followed screening was narrowed down by manually selecting those ubiquitin like domains that end on the motif of X-X-[R/A/E/K]-X-X-[G/X]-G. Eight candidates were finally selected (Table 1) with four candidates containing X-X-R-X-X-[G/X]-G motif and the other four containing the X-X-K-X-X-[G/X]-G motif. Seven are human proteins and one is from* Drosophila*.

### 3.2. ATP-PPi Assay and Kinetics of Short Peptides with Ube1

We synthesized C1-C8 peptides (7mers based on the motif) and ascertained whether they were reactive with human E1 Ube1. For comparison, we used the C-terminal sequence of wild-type ubiquitin (P1) as negative control and the short peptides P3 (VQRYWGG) and P4 (VYRFYGG) we previously reported as positive controls [12].

**Figure 1:**
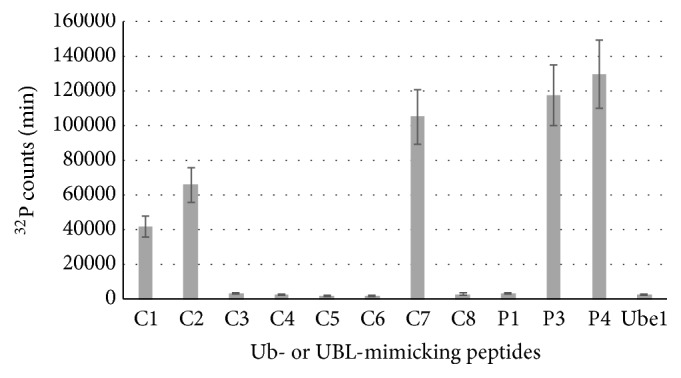
ATP-PPi exchange rates of peptides with Ube1. ^*C1*^*P*<0.05 versus P1, ^*C2*^*P*<0.05 versus P1, and ^*C7*^*P*<0.05 versus P1, n=3.

We measured the reactivity of the peptides with Ube1 by ATP-PPi exchange assay (Figure 1). P1 peptide could not be conjugated to Ube1, whereas both P3 and P4 showed significant activity with Ube1 as we have previously reported [12]. Among the eight short peptides we tested, only C1, C2, and C7 displayed affinity with Ube1 with a similar level of efficiency as P3 and P4. All three peptides share the same sequence of X-X-R-X-X-G-G. We concluded that the Arg and Gly-Gly residues are indispensable for E1 recognition. In comparison, neither C8, containing Arg but only one Gly, nor C3, which possesses the C-terminal Gly-Gly but with a homologous replacement of Arg to Lys, showed any reactivity toward Ube1.

We further measured the kinetic parameters for the peptides C1, C2, C7, P1, P3, and P4 by ATP-PPi assays (Table 2). The results showed the activity of these peptides was 67-192-fold higher than that of P1 peptide. In particular, C7 exhibited the highest activity of the three peptides identified in this study.

### 3.3. Peptides on Native Frames

We speculated that the parental proteins of C1, C2, and C7 could potentially be activated by Ube1. The encoding genes of the proteins refer to candidates 1, 2, and 7 which were synthesized. We did not use the full length proteins but truncated the proteins after the Gly-Gly residue in the middle due to the importance of the free C-terminal Gly-Gly for the UBLs.

**Figure 2:**
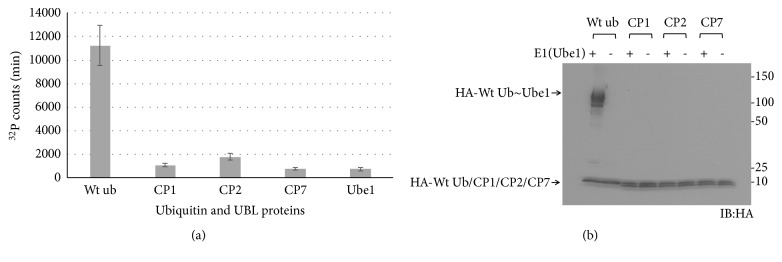
(a) ATP-PPi exchange rates of wild-type ubiquitin and CP1, CP2, and CP7 proteins with Ube1. ^*CP1*^*P*<0.05 versus wt ub, ^*CP2*^*P*<0.05 versus wt ub, and ^*CP7*^*P*<0.05 versus wt ub, n=3. (b) Western-blot of E1 transfer of wild-type ubiquitin and CP1, CP2, and CP7 proteins with Ube1.

We fused an HA-tag at the N-termini of these truncated candidate proteins and expressed them in* E. coli *cells. The native protein sequences used here are listed in Figure S1. These recombinant proteins were named as CP1, CP2, and CP7. ATP-PPi assays revealed all three proteins exhibited significantly lower binding affinity for Ube1 although they contain the hypothetical UBL motifs (Figure 2(a)). Western-blotting further confirmed that none of these candidate proteins was reactive with Ube1 (Figure 2(b)). Meanwhile these truncated proteins could not bind to Nedd8 E1 (NAE) or SUMO E1 (SAE) (data not shown). These data suggested that CP1, CP2, and CP7 may not be potential ubiquitin like proteins although the free peptides could be activated by Ube1. Another reason is Ube1 may not be the cognate E1 of these candidate proteins.

### 3.4. Peptide on Ubiquitin Frame

**Figure 3:**
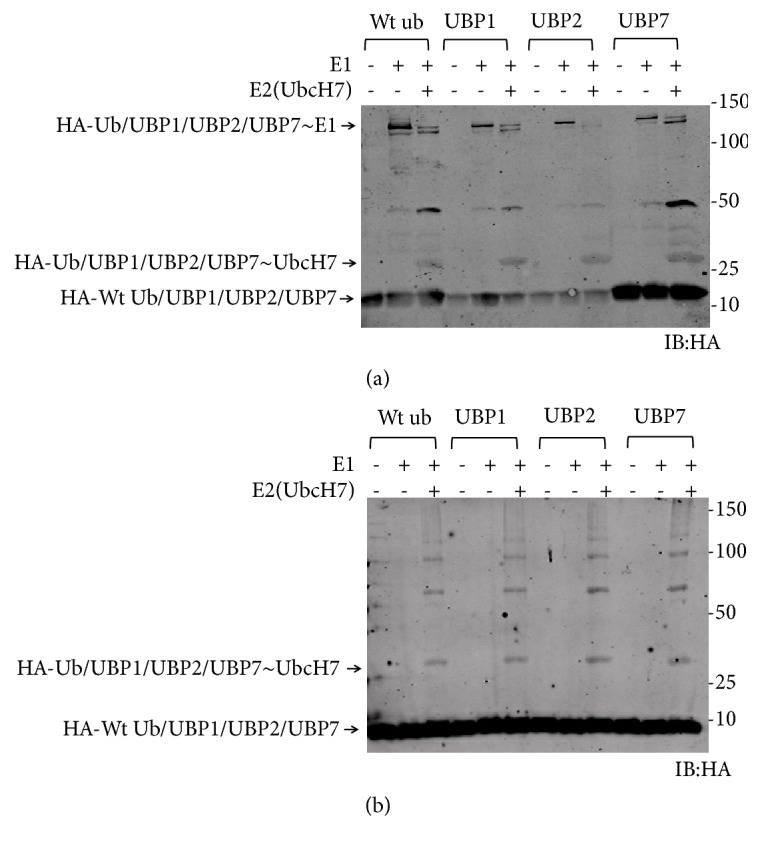
Transfer of wild-type ubiquitin and ubiquitin mutants to Ube1 and UbcH7. (a) Reducing SDS-PAGE. (b) Nonreducing SDS-PAGE.

We have previously reported that replacing the C-terminal sequence of ubiquitin by other peptides containing the motif V-X-R-X-X-X-G would not impact the binding capacity of ubiquitin mutants with E1 and E2 enzymes [11]. Here we similarly replaced the C-terminal peptide (P1) in wild-type ubiquitin by one of C1, C2, and C7 peptides, which led to the generation of the ubiquitin mutants UBP1, UBP2, and UBP7. Western-blotting confirmed these ubiquitin mutants could be activated effectively by Ube1 and UbcH7 as wild-type ubiquitin under reducing SDS-PAGE conditions (Figure 3(a)). E1 bands could not be seen under nonreducing SDA-PAGE conditions but the E2-ubiquitin or E2-ubiquitin mutant conjugates were clearly visible (Figure 3(b)). Taken together, these results suggested that ubiquitin and the three ubiquitin mutants were all capable of forming thioester intermediates with Ube1 and UbcH7. We did not perform E3 transfer because our previous study manifested that E3 enzymes will block the transfer of ubiquitin mutants with the C-terminal tail replacement except the mutations which just happened at Leu73 residue [11].

### 3.5. Peptide on PCP Frame

**Figure 4:**
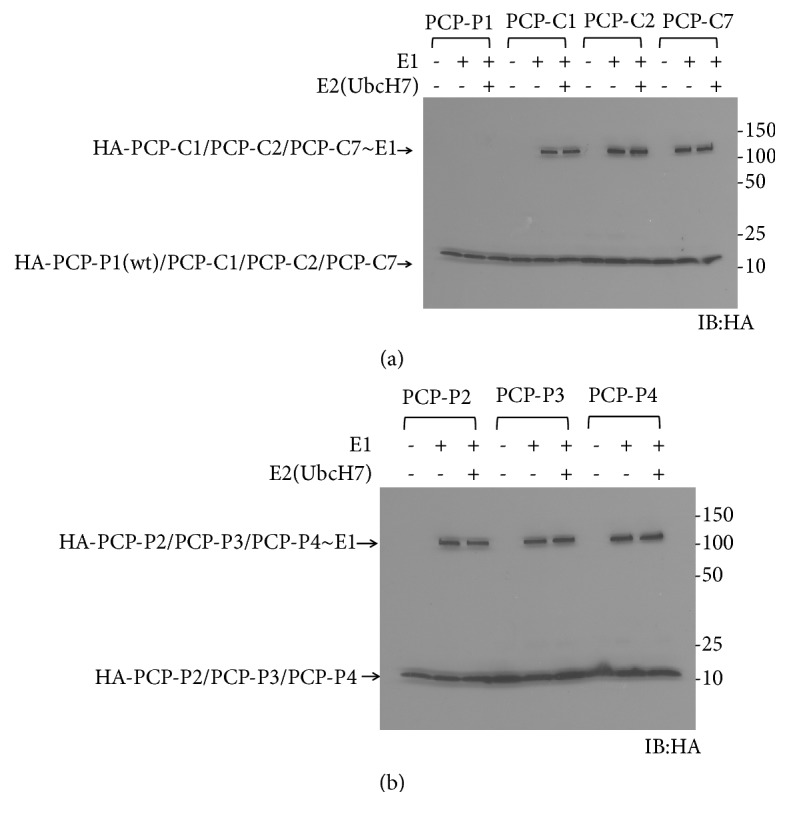
(a) Transfer of PCP-peptides to Ube1 and UbcH7. (b) Transfer of PCP-P2, PCP-P3, and PCP-P4 to Ube1 and UbcH7.

**Figure 5:**
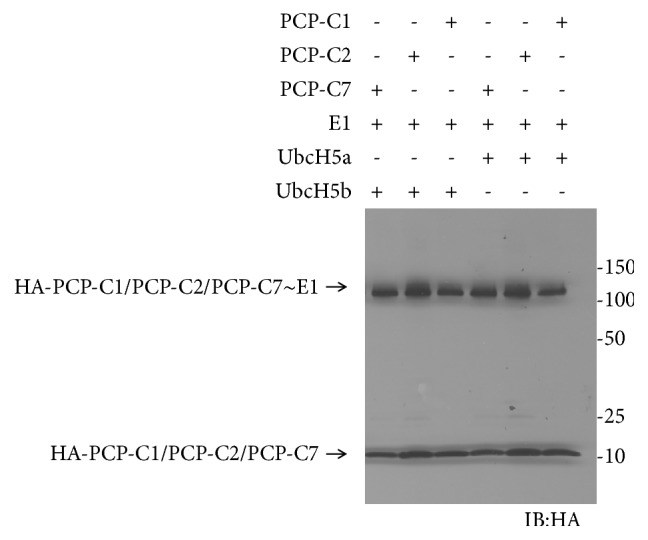
Transfer of PCP-peptides to Ube1 and E2 enzymes UbcH5a and UbcH5b.

Our previous studies have demonstrated that fusion with PCP (peptidyl carrier protein) could increase the expression of the target protein without affecting its activity [11]. We thus fused C1, C2, and C7 peptides as the C-terminal tails with a PCP protein via a (GGGGS)_3_ linker and expressed the recombinant proteins in* E. coli* cells. We examined the reactivity of the PCP-peptide fusion with Ube1 and UbcH7. We tested PCP-P1 as a negative control and found PCP-P1 could not be activated by either Ube1 or UbcH7 (Figure 4(a)). Meanwhile, PCP-C1, PCP-C2, and PCP-C7 all exhibited significant reactivity toward Ube1. However, we did not see any PCP-peptide fusion could be transferred to E2 enzyme UbcH7. To verify if PCP-C1, PCP-C2, and PCP-C7 are unreactive with E2, we constructed PCP-P2, PCP-P3, and PCP-P4 as controls. Our previous study showed free peptides P2, P3, and P4 could be transferred to E2 and E3 [12]. Similarly, PCP-P2, PCP-P3, and PCP-P4 were able to be transferred to Ube1 but not UbcH7 (Figure 4(b)). Additional transfer experiments were performed with other E2 enzymes such as UbcH5a and UbcH5b and produced similar results (Figure 5). We found all the E2s rejected the transfer of the PCP-peptide fusions although these fusions could been transferred to Ube1, suggesting that the recognition of E2 with peptide-fused proteins might depend on the overall structure of the frame fused with peptide.

## 4. Discussion

In eukaryotic cells, ubiquitin and ubiquitin like proteins (UBLs) are covalently attached to other cellular proteins to modify the functions of these substrates. Although the sequences are not highly similar, all the UBLs possess the same three-dimensional structure: *β*-grasp fold (ubiquitin fold) and a flexible C-terminal tail terminating with a Gly [17, 18]. Usually, each UBL possesses its own set of enzymes. Nedd8, the closest relative of ubiquitin, can be activated by ubiquitin E1 Ube1 and enters the ubiquitin E1-E2-E3 cascades [19, 20]. The ubiquitin like protein FAT10 (HLA-F adjacent transcript 10) is activated by ubiquitin E1 Uba6 and further transferred to Uba6 specific E2 Use1 [21–23]. Atg8 and Atg12 share one E1 Atg7 and are transferred to different E2 enzymes, respectively [24].

Ubiquitin and most of UBLs are synthesized as inactive precursors which need to be cleaved at their C-termini to expose the Gly residue that is the active site for substrates conjugation. Some proteases called deubiquitinating enzymes (DUBs) are responsible for the processing of ubiquitin [25], whereas other similar specific proteases can process the precursors of UBLs (UBL-specific proteases, ULPs) [18].

In this study, we searched the proteins with both *β*-grasp fold and the C-terminal X-X-[R/A/E/K]-X-X-[G/X]-G motifs in UNIPROT sequence database. Finally, eight candidate proteins were selected as targets for further study. We first synthesized eight short peptides based on the motif in selected candidates and examined their reactivity toward Ube1. Three peptides that contain X-X-R-X-X-G-G sequences were reactive with Ube1. Our previous work showed Arg residue and Gly-Gly tail at the C-terminus of ubiquitin are the most important features for the E1 activation [11]. We further investigated if these reactive peptides could be transferred to E1 and E2 enzymes when they attached on the C-terminus of a given protein. We fused the C-terminal peptides with the native protein frames but did not see any reactivity between the native proteins with Ube1. We also tested the reactivity of the native proteins with Nedd8 E1 NAE and SUMO E1 SAE and neither of the E1 could activate these proteins (data not shown). These results suggested that the native proteins might not be UBLs although they share both *β*-grasp fold and C-terminal X-X-[R/A/E/K]-X-X-[G/X]-G motif. Another reason is that their E1s are undiscoverable.

The three reactive peptides showed good transfer to E1 and E2 enzymes on the ubiquitin frame. We replaced the C-terminal peptide of ubiquitin by these peptides and found the new ubiquitin mutants were formed thioester intermediates with Ube1 and UbcH7.

An interesting result is when these C-terminal peptides were fused with a PCP protein, the transfer of PCP-peptide fusions was blocked by all the E2 enzymes, although they could still be activated by Ube1. Here we put the PCP before the N-termini of the reactive peptides and make the PCP-peptide fusions because in our previous study we fused PCP at the N-terminus of ubiquitin and found the PCP-ubiquitin fusion could be transferred to both Ube1 and UbcH7 (data not shown). As the free peptides could be transferred to UbcH7, we speculated that the PCP frame blocked the interaction of PCP-peptide fusions with E2. The transfer of peptide-fused proteins to E2 enzymes depended not only on the sequences of the peptides, but also on the overall structures of the proteins fused with the peptides. Further investigations on how the different protein frames impact the interaction of E2 and how the E2 enzymes recognize the different length of ubiquitin-mimicking peptides are ongoing.

## 5. Conclusion

Eight peptides were selected from database based on the structures and conserved motif. Three of them showed reactivity with ubiquitin E1 enzyme Ube1. These reactive peptides could be transferred to E1 and E2 on a ubiquitin frame but not to E2 when fused with a PCP protein, suggesting the E2 transfer for the fusions of ubiquitin-mimicking peptides with proteins required the proper structures of the fused proteins.

## Figures and Tables

**Table 1:** Eight candidate proteins selected with UBL motifs.

Candidate	Candidate name	Motif sequence
C1	Human C1orf55	EPRLCGG
C2	*Drosophila* homologue of C1orf55	VLRQLGG
C3	Human splicing factor 3A subunit 1 isoform 1	ALKERGG
C4	USP15 protein (Homo sapiens)	EQKNEDG
C5	Protein DDI1 homolog 1	LQKDNVG
C6	Ubiquilin-like protein (Homo sapiens)	VIKSKQG
C7	Ubiquitin-like protein fubi and ribosomal protein S30 precursor	AGRMLGG
C8	Ubiquitin-fold modifier 1 (Homo sapiens)	IPRDRVG
P1	Ubiquitin	VLRLRGG

**Table 2:** Kinetic parameters of ATP-PPi exchange catalyzed by Ube1 and peptides. ^*a*^*P*<0.05 versus Ube1+P1, ^*b*^*P*<0.05 versus Ube1+P1, and ^*c*^*P*<0.05 versus Ube1+P1, n=3.

	K_m_	k_cat_	k_cat_/K_m_
	(*μ*M)	(min^−1^)	(*μ*M min^−1^)
Ube1+ P1	ND	ND	5.24x 10^−5^
Ube1+C1	651.4±49.3^a^	2.29±0.59^a^	3.52 x10^−3^^a^
Ube1+C2	611.1±23.1^b^	3.73±0.87^b^	6.10 x10^−3^^b^
Ube1+C7	542.6±122.1^c^	5.54±1.37^c^	1.01 x10^−2^^c^
Ube1+ P3	476.1±163.2	6.68±0.77	1.40 x10^−2^
Ube1+ P4	250.9±55.6	13.98±2.08	5.57x10^−2^

## Data Availability

The data used to support the findings of this study are available from the corresponding author upon request.

## Supplementary Materials

Supplementary Materials Supplementary Materials including the full length sequences of candidates 1, 2, and 7 can be found with this paper.
