# Supplementary material for: Ubiquitin-Mimicking Peptides Transfer Differentiates by E1 and E2 Enzymes
Source: Biomed Res Int. 2018 Aug 30;2018:6062520. doi: 10.1155/2018/6062520 (PMC6136576; doi:10.1155/2018/6062520)
Supplement: Supplementary Materials — including the full length sequences of candidates 1, 2, and 7 can be found with this paper. [file 6062520.f1.docx]

**Supplementary Materials**

1. UBL candidates 1 (Human C1orf55)

MAEAAALVWIRGPGFGCKAVRCASGRCTVRDFIHRHCQDQNVPVENFFVKCNGALINTSDTVQHGAVYSL**EPRLCGG**KGGFGSMLRALGAQIEKTTNREACRDLSGRRLRDVNHEKAMAEWVKQQAEREAEKEQKRLERLQRKLVEPKHCFTSPDYQQQCHEMAERLEDSVLKGMQAASSKMVSAEISENRKRQWPTKSQTDRGASAGKRRCFWLGMEGLETAEGSNSESSDDDSEEAPSTSGMGFHAPKIGSNGVEMAAKFPSGSQRARVVNTDHGSPEQLQIPVTDSGRHILEDSCAELGESKEHMESRMVTETEETQEKKAESKEPIEEEPTGAGLNKDKETEERTDGERVAEVAPEERENVAVAKLQESQPGNAVIDKETIDLLAFTSVAELELLGLEKLKCELMALGLKCGGTLQERAARLFSVRGLAKEQIDPALFAKPLKGKKK

1. UBL candidates 2 (*Drosophila* homologue of C1orf55)

MGINIFINSKYIISCGDHIKYNELYSRIAEKTNLQPEEYYLVSNGKRLEEEIPSGDVHC**VLRQLGG**KGGFGSMLRAIGAQIEKTTNREACRDLSGRRLRDINEEKRVRAWLEKQGEREREAEERKKRKIEKLLAVPKHDFKDEKYDEARANLTEKVNDAFEEGLKQAEENKEKGVEEATSSGTKRKSPAVDKTKAKKKKKGTLWIGDDISGSDSDSDDSEEEEPKTQKKAIQN

1. UBL candidates 7 (Ubiquitin-like protein fubi and ribosomal protein S30 precursor [Homo sapiens])

MQLFVRAQELHTFEVTGQETVAQIKAHVASLEGIAPEDQVVLLAGAPLEDEATLGQCGVEALTTLEV**AGRMLGG**KVHGSLARAGKVRGQTPKVAKQEKKKKKTGRAKRRMQYNRRFVNVVPTFGKKKGPNANS

Fig S1. Protein sequences of the full length candidates. The truncated sequences of these candidates (underscored) were expressed and used in this paper. 7-mer peptides of each candidate are bold font in black. A. Candidate1, B. Candidate 2, C. Candidate 7.
